# Guards and Culprits in the Endoplasmic Reticulum: Glucolipotoxicity and ****β****-Cell Failure in Type II Diabetes

**DOI:** 10.1155/2012/639762

**Published:** 2011-10-01

**Authors:** Udayakumar Karunakaran, Han-Jong Kim, Joon-Young Kim, In-Kyu Lee

**Affiliations:** Departments of Internal Medicine, Biochemistry and Cell Biology, Research Institute of Aging and Metabolism and World Class University Program, Kyungpook National University School of Medicine, Daegu 700-721, Republic of Korea

## Abstract

The endoplasmic reticulum (ER) is a cellular organelle responsible for multiple important cellular functions including the biosynthesis and folding of newly synthesized proteins destined for secretion, such as insulin. The ER participates in all branches of metabolism, linking nutrient sensing to cellular signaling. Many pathological and physiological factors perturb ER function and induce ER stress. ER stress triggers an adaptive signaling cascade, called the unfolded protein response (UPR), to relieve the stress. The failure of the UPR to resolve ER stress leads to pathological conditions such as *β*-cell dysfunction and death, and type II diabetes. However, much less is known about the fine details of the control and regulation of the ER response to hyperglycemia (glucotoxicity), hyperlipidemia (lipotoxicity), and the combination of both (glucolipotoxicity). This paper considers recent insights into how the response is regulated, which may provide clues into the mechanism of ER stress-mediated *β*-cell dysfunction and death during the progression of glucolipotoxicity-induced type II diabetes.

## 1. Introduction

Type II diabetes is a heterogeneous syndrome resulting from progressive impairment of *β*-cell insulin secretion and insulin resistance in target tissues. Prevalence of type II diabetes, in which the body attempts to compensate for insulin resistance by augmenting insulin secretion, has increased because of the rising rate of obesity [1]. However, it is increasingly clear that pancreatic *β*-cell dysfunction also contributes to type II diabetes [2]. Healthy pancreatic *β*-cells display a dramatic response to nutrients and to obesity-associated insulin resistance through hypersecretion of insulin to maintain energy homeostasis. But, in type II diabetes, *β*-cells are unable to sustain a compensatory response, leading to *β*-cell dysfunction and death [3]. Although the cause of the metabolic deterioration is unknown, several hypotheses have been proposed, including mitochondrial dysfunction, oxidative stress, ER stress, hyperglycemia (glucotoxicity), dyslipidemia (lipotoxicity), and the combination of both (glucolipotoxicity) [4–7]. 

Recent studies have suggested that elevated glucose, along with circulating free fatty acids (FFAs), and particularly those originating from intra-abdominal fat stores are the major culprits in insulin resistance and *β*-cell dysfunction [6, 7]. The chronic hyperglycemia and hyperlipidemia cause combined, detrimental effects defined as glucolipotoxicity on *β*-cell function and survival [7]. However, the underlying molecular and cellular mechanisms by which glucolipotoxicity contributes to *β*-cell dysfunction and death in type II diabetes remain under debate. A recent observation based on experimental, clinical, and genetic evidence suggests that the endoplasmic reticulum (ER) may be responsible for the molecular mechanisms of glucolipotoxicity contributing to *β*-cell dysfunction in type II diabetes [8, 9]. In this paper, we discuss the involvement of ER in glucolipotoxicity-induced *β*-cell dysfunction and death along with the involvement of mitochondria.

## 2. ER Stress Response

Pancreatic *β*-cells display a marked response to nutrient signals through balance between the anabolic hormone insulin and the catabolic hormone glucagon, which are used to maintain energy homeostasis. To mount an appropriate response, pancreatic *β*-cells require suitable sensors and signaling molecules, which integrate these signals to modulate insulin secretion to maintain homeostasis. 

The ER is an integral contributor to protein synthesis, folding, maturation, trafficking, and degradation, and it may be an ideal site for nutrient sensation at the subcellular level [10]. The quality control machinery of the ER operates via specialized proteins and a specific chemical environment to ensure proper folding and processing of secretory proteins, and degradation of misfolded/unfolded proteins, including insulin, to maintain glucose homeostasis. However, overloading this machinery causes the more accumulation of misfolded/unfolded proteins in the ER and reduces the quality and quantity of ER, leading to ER stress [11]. To cope with this condition, cells activate an adaptive system linking the ER lumen with the cytoplasm and nucleus called the unfolded protein response (UPR). The UPR restores ER homeostasis, by attenuating global protein translation to decrease the protein overload and by increasing the expression of genes that induce protein folding and also promote ER-associated protein degradation (ERAD) to remove misfolded proteins [12]. 

The three branches of the UPR are mediated by the ER-membrane associated proteins PERK (protein kinase R-like ER kinase), IRE1*α* (Inositol requiring enzyme 1), and ATF6 (activating transcription factor 6). Under unstressed conditions, these three proteins are held by the abundant ER chaperone Bip/glucose-regulated protein 78 (GRP78) at the N-terminal domains of PERK and IRE1*α* and at the carboxyl terminal of ATF6, preventing their aggregation and rendering them inactive [13]. Under ER stress conditions, PERK is autophosphorylated and in turn phosphorylates serine 51 of eukaryotic translation initiation factor 2*α* (eif2*α*), rendering it unable to efficiently initiate translation, leading to global inhibition of protein synthesis and at the same time inducing translation of the transcription factor ATF4. ATF4 protein translocates to the nucleus and upregulates ER stress target genes, including C/EBP homologous protein (CHOP) and downstream growth arrest and DNA damage-inducible protein (GADD34) that acts as a nonenzymatic cofactor for protein phosphatase-1 (PP-1), leading to eif2*α* dephosphorylation for translational recovery [14, 15]. 

Under ER stress condition, activation of ATF6 involves the dissociation of Bip/GRP78 from its luminal domain and translocation to the Golgi for proteolytic processing where it is cleaved into its active form, which translocates into the nucleus to induce chaperon protein genes such as Bip/GRP78, GPR94, and calreticulin to enhance protein folding [16]. IRE1*α*, the third ER stress sensor, is a type 1 transmembrane protein with endoribonuclease activity. Similar to PERK, IRE1*α* is autophosphorylated in response to accumulation of misfolded/unfolded proteins in the ER. Once activated, IRE1*α* catalyzes the splicing of X-box-binding protein 1 (XBP-1) mRNA, leading to translation of the active transcription factor XBP-1 that induces the expression of genes required for protein folding, ER to Golgi transport, and endoplasmic-reticulum-associated protein degradation (ERAD) [17].

## 3. UPR under Glucolipoadaptation in *β*-Cells

Adaptation to metabolic changes requires regulation and coordination of many homeostatic systems, since the quality and quantity of available nutrients does not temporally match cellular needs. Acute exposure of *β*-cells to high glucose induces mild UPR signaling accompanied by phosphorylation and activation of IRE1*α*, leading to glucose-induced insulin biosynthesis [18]. Conversely, inactivation of IRE1*α* signaling by siRNA or inhibition of IRE1*α* phosphorylation hinders glucose-induced insulin biosynthesis, indicating that acute IRE1*α* activation is required for proinsulin biosynthesis. Surprisingly, however, acute high glucose-induced IRE1*α* was found not to splice the downstream target XBP-1, implying that IRE*α*-mediated XBP-1 splicing is not essential for proinsulin biosynthesis. 

Another study suggested that transient high glucose upregulates the ER resident protein oxidoreductase 1*α* (ERO1*α*), an activator of protein disulfide isomerase (PDI), which plays an important role in disulfide bond formation. Thus, ERO1*α* may activate insulin biosynthesis by enhancing disulfide bond formation in proinsulin in the ER [19]. Rutkowski and Kaufman suggested that eif2*α* phosphorylation limits proinsulin mRNA translation under low-glucose condition [10]. However, paradoxically, eif2a phosphorylation seems to be needed to upregulate proinsulin mRNA translation to compromise the uncontrolled insulin translation in response to physiologic intermittent high glucose levels [11]. Steady-state eif2*α* phosphorylation in glucose-induced protein translation is short, and it can be rapidly dephosphorylated by physiological stimuli via a signaling pathway that activates GADD34 and PPI [20]. 

In parallel with glucose, saturated and unsaturated FFAs elicit quantitatively and qualitatively different ER stress signaling in *β*-cells. Most investigators have employed palmitate or oleate as the fatty acids of choice because they represent the major species to which *β*-cells might be exposed *in vivo* [21]. However, considering the specific effects of fatty acids on *β*-cell viability, palmitate is more potent than oleate in triggering ER stress in clonal and primary rodent *β*-cells, and in human islets [22–24]. Exposure to long-chain FFAs within the physiologic range can directly stimulate insulin secretion through changes in ER calcium (Ca^2+^) handling [25]. ER is thought to be the main dynamic intracellular Ca^2+^ storage compartment in *β*-cells. Palmitate causes increases in phosphorylation of the PERK branch and IRE1*α*-activated spliced form of XBP-1, but the effects of oleate are much less significant [26]. The marked activation of PERK-eif2*α* phosphorylation by palmitate leads to the induction of ATF4 and CHOP expression, which results in inhibition of protein translation [27]. Thus, FFAs differentially regulate the UPR response under physiological conditions to maintain homeostasis. Growing evidence supports the notion that early activation of UPR signaling improves *β*-cell homeostasis in glucolipoadaptation.

## 4. ER Stress under Glucolipotoxicity

Accumulating evidence suggests that glucolipotoxicity contributes to *β*-cell dysfunction during the development of type II diabetes. Chronic exposure of *β*-cells to supraphysiological levels of glucose or FFAs has been shown to be cytotoxic and causes *β*-cell dysfunction and failure [28–30]. Briaud et al. have provided evidence that lipotoxicity occurs in the presence of concomitantly elevated levels of glucose [31]. Several mechanisms have been proposed for glucolipotoxicity-induced *β*-cell dysfunction and failure, such as increased ROS, ceramide, and nitric oxide levels, and mitochondrial perturbations [32–34]. Recent evidence suggests that ER stress is linked to insulin resistance in diabetes and also expansion of ER was detected in *β*-cells from type II diabetic patients [35, 36]. Furthermore, increased expression of ER stress markers has been demonstrated in db/db mouse islets and *β*-cells of type 2 diabetes patients [23, 36]. These findings suggest that ER stress may be a pathophysiological event responsible for *β*-cell dysfunction and failure in type II diabetes. 

Endoplasmic reticulum Ca^2+^ is an important signaling molecule in the *β*-cell, and fluctuations in Ca^2+^ levels in the ER can affect many functions of the endoplasmic reticulum, including protein synthesis, processing, and interchaperone interactions [37]. However, experimental data suggest that saturated and, to a lesser extent, unsaturated FFAs, trigger the ER stress response through depletion of ER Ca^2+^ stores [26, 38, 39]. Several discrepancies appear in studies of FFA-induced ER Ca^2+^ depletion, but the mechanism appears to be similar to the direct effects on sarcoplasmic-endoplasmic reticulum Ca^2+^ ATPase-2b (SERCA) pump activity [39]. ER Ca^2+^ depletion affects protein folding in the ER, because high luminal Ca^2+^ is essential for proteolytic processing and folding of proinsulin [37]. In addition, palmitate causes rapid redistribution and degradation of carboxypeptidase E (CPE) by depleting ER Ca^2+^. CPE is a soluble membrane-bound enzyme in secretory granules involved in insulin processing. Degradation of CPE by palmitate was found to cause accumulation of unprocessed proinsulin in the secretory pathway [40]. Along with depletion of ER Ca^2+^, palmitate also hampers ER-to-Golgi trafficking, as monitored using a temperature-sensitive vesicular stomatitis virus G protein, contributing to the accumulation of misfolded proteins and impacting ER integrity and function [41].

## 5. UnfolDE(A)D Protein Response

As described above, elevated glucose and FFA act synergistically in causing pleiotropic effects leading to *β*-cell decompensation and apoptosis during type II diabetes. It has been shown that palmitate induces *β*-cell dysfunction and apoptosis via activation of ER stress. This activates the UPR to restore normal ER function; when the UPR fails to adequately restore ER function, it turns on signaling pathways leading to apoptosis [42, 43]. The ER-localized protein Bip/GRP78 is a multifunctional chaperone and sensor of protein misfolding and controls activation of the UPR response in ER stress. Bip/GRP78 is upregulated during ER stress; overexpression of Bip/GRP78 under hyperglycemic conditions improves insulin levels and *β*-cell function [44]. In mouse MIN6 cells, Bip/GRP78 overexpression reduces ER stress and partially protects cells against fatty acid-induced apoptosis [23]. C/EBPbeta, a CCAAT/enhancer-binding protein (C/EBP) family basic leucine zipper (bZip) transcription factor, was found to be increased in diabetic islets and to block the induction of Bip/GRP78 due to the suppressed transactivation of ATF6*α*, thereby increasing the vulnerability of *β*-cells to ER stress [45]. However, the role of Bip/GRP78 under palmitate-induced ER stress is under debate. In BRIN-BD11 cells, palmitate exposure does not induce Bip/GRP78 [46]. These discrepancies may be dependent on cell type, and a direct comparison between studies would be required to understand the exact phenomenon that happened under these experimental conditions. 

As with Bip/GRP78, the effects of palmitate on the ATF6*α* pathway in *β*-cells are also controversial. Transient transfection of INS-1 cells with HA-tagged ATF6*α* revealed that ATF6*α* protein was distributed around the nucleus and in the periphery of the cell in response to palmitate without induction of Bip/GRP78, but not in control or oleate-treated cells [47]. By contrast, Kharroubi et al. showed induction of the ATF6*α*-GPR78 signaling pathway by palmitate in INS-1 cells [48]. Recent evidence has also shown that missense mutations and polymorphisms within ATF6*α* may be linked to type II diabetes [49, 50]. Therefore, further studies are in need to delineate the exact ATF6*α* signaling pathways induced by palmitate in *β*-cell failure.

By contrast, mice with a PERK deletion develop diabetes within a few weeks of birth due to progressive *β*-cell loss, highlighting the importance of the PERK-mediated ER stress response in the regulation of *β*-cell function and survival [51]. Like other ER sensors, PERK is maintained in an inactive state by binding to Bip/GRP78. Once activated, PERK autophosphorylates and catalyzes the phosphorylation of eif2*α* [52, 53]. This results in a general attenuation of translation. Several studies have demonstrated that palmitate and, to a lesser extent, oleate activate rapid phosphorylation of the PERK by depletion of ER calcium leading to phosphorylation of eif2*α*, resulting in an overall decrease in translation, but increased translation of selected proteins including ATF3, ATF4, and CHOP [23, 39, 47, 54]. Induction of ATF4 by upstream PERK-eif2*α* leads to the induction of CHOP via binding of ATF4 to the C/EBP-ATF binding site in the CHOP promoter [55]. Induction of ATF3, a proapoptotic protein, by palmitate leads to *β*-cell apoptosis [56]. In addition, it has been shown that ATF3 downregulates the expression of IRS-2 in *β*-cells [57]. By contrast, knockdown of ATF3 was found to increase palmitate-induced apoptosis instead of protecting against apoptosis [56]. Recently, Zmuda et al. showed that ATF3 knockout mice fed a high-fat diet for 12 weeks had significantly reduced serum insulin levels without insulin sensitivity being affected and without *β*-cell apoptosis being induced [58]. So far, the downstream targets of ATF3 in *β*-cells are not well known and further investigation is needed to clarify these unexpected findings. 

Expression of CHOP induced by palmitate induces cell death through transcriptional regulation of survival and death effectors. CHOP was found to be localized in the nucleus as opposed to the cytoplasm in pancreatic sections from diabetic patients, suggesting that CHOP nuclear translocation is a discrete and necessary step for apoptosis induction [59]. CHOP also downregulates the expression of the antiapoptotic protein Bcl-2 and increases cellular reactive oxygen species, which likely contribute to ER stress-associated cell death [60]. CHOP also upregulates expression of ERO1*α*, an ER oxidase, causing hyperoxidizing conditions in the ER leading to apoptosis [61, 62]. 

**Figure 1:**
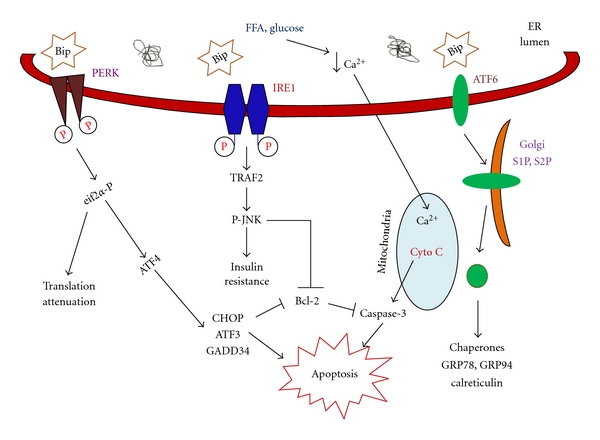
FFA-induced endoplasmic reticulum stress transduction and apoptosis in pancreatic *β*-cells, and its mechanisms; how FFA elicits *β*-cell apoptosis is discussed in detail in the text.

Growing evidence shows that the IRE1*α*-XBP-1 pathway is activated in *β*-cells by palmitate treatment, but that it is less sensitive to the monounsaturated fatty acid oleate [46, 47, 54]. Oleate can efficiently counteract palmitate-induced XBP-1 mRNA splicing, and knockdown of XBP-1 was shown to potentiate oleate- but not palmitate-mediated *β*-cell apoptosis, suggesting differential activation of pro- and antiapoptotic signals by downstream of IRE1*α* [63]. In this regard, IRE1*α* recruits the adaptor protein TNF receptor-associated factor 2 (TRAF2) to the ER membrane, leading to activation of JNK and downstream proapoptotic signaling [64]. In rodent *β*-cells, the association of IRE1*α* and TRAF2 contributes to ER-triggered apoptosis [65, 66]. Increased levels of saturated FFAs lead to JNK activation, IRS1 and IRS2 ser/thr phosphorylation, and downregulation of insulin signaling and gene expression [67]. Furthermore, inactivation of JNK in *β*-cells prevents palmitate-induced inhibition of insulin gene expression [68]. In addition, blockade of palmitate-induced activation of JNK using a JNK inhibitor partially protected *β*-cells from the effects of palmitate [64]. However, the downstream mechanism by which JNK leads to apoptosis is not clear, and this may be mediated via caspase activation, which is described in the next section (Figure 1). 

## 6. ER is Linked to Mitochondria to Induce Apoptosis

The ER and mitochondria are capable of modifying their structure and function in response to changing environmental challenges. These two organelles form a highly dynamic interconnected network for activating apoptosis [69]. However, the regulatory mechanisms, which determine cell status leading to cell survival or cell death in response to ER stress, have not been well established. Mitochondria have a heterogeneous shape among different cell types and their ability to effectively function is influenced by their dynamic behavior. Interestingly, mitochondrial shape and morphology are determined by two dynamically opposed processes: fusion and fission. Ablation of both fusion and fission produces a profound effect on the progression of cells to apoptosis [70]. It has been shown that the mitochondria of *β*-cells from Zucker diabetic rats are fragmented, suggesting an imbalance in the regulation of mitochondrial fusion and fission [71]. Under normal conditions, mitochondria in *β*-cells continuously undergo fusion and fission and these interactions may function to negate the detrimental effects of long-term exposure of *β*-cells to palmitate under high-glucose conditions, causing mitochondrial fragmentation and impairing network dynamics by abolishing fusion and fission activity. However, in the absence of palmitate, high glucose does not affect mitochondrial architecture [72]. 

It has been clearly demonstrated that apoptosis induction leads to fragmentation of the mitochondrial network. Moreover, inhibition of mitochondrial fission by Fis1, an outer mitochondrial membrane fission protein that determines the frequency of mitochondrial fission, reduced apoptosis in *β*-cells [72]. This has been questioned by another study, which ruled out the possibility that fragmentation occurs early in the cell death pathway [73]. These conflicting results may be reconciled by the fact that mitochondria are able to receive different stress signals and integrate them. How, then, do high-fat glucose conditions promote mitochondrial fragmentation and apoptosis? It is assumable that crosstalk between the ER and mitochondria may determine cellular commitment to apoptosis through Ca^2+^. Exposure of *β*-cells to high-fat glucose conditions causes release of Ca^2+^ from the ER to the cytoplasm, leading to a rise in cytosolic Ca^2+^ concentration that reflects increased mitochondrial Ca^2+^ uptake. Increased mitochondrial Ca^2+^ uptake enhances local buffering capacity and release of proteins capable of apoptosis activation [74]. Subsequently, Ca^2+^ activates the phosphatase calcineurin, which dephosphorylates and inactivates dynamin-related protein 1 (Drp1), a master regulator of mitochondrial fission. Interestingly, inhibition of calcineurin activation partially prevented palmitate-induced apoptosis [75]. In addition, palmitate-induced activation of mitochondrial transition pores caused depolarization of the mitochondrial inner membrane and cytochrome c release into the cytosol, which stimulates the assembly of the apoptosome leading to activation of caspase-9, which, in turn, activates caspase-3, leading to DNA fragmentation and cell death [63, 64, 76, 77]. Moreover, mitochondrial cytochrome c translocates to the ER, where it selectively binds to InsP3R, leading to a sustained rise in cytosolic Ca^2+^ [78]. 

In addition, the Bcl-2 family of proteins has a role in mediating ER stress-induced apoptosis. Under normal conditions, Bcl-2 can bind and sequester BH3-only proteins, preventing these proteins from triggering oligomerization and activation of Bax and Bak [79]. Upon stimulation, Bax translocates from the cytosol to the mitochondria and oligomerizes with Bak, resulting in mitochondrial outer membrane permeabilization and release of cytochrome c into the cytosol [80, 81]. Mcl-1, a member of the antiapoptotic Bcl-2 protein family, prevents Bax activation and translocation by sequestering factors contributing to Bax translocation [81]. Allagnat et al. recently demonstrated that, under lipotoxic conditions, Mcl-1 expression was downregulated in *β*-cells leading to translocation of Bax into mitochondria, cytochrome c release, and caspase-3 cleavage and apoptosis [82]. However, supporting this concept, another study has suggested the involvement of translationally controlled tumor protein (TCTP) with no sequence similarity to any other known protein. TCTP binds to antiapoptotic proteins in the Bcl-2 family, such as Mcl-1 and Bcl-XL, and antagonizes apoptosis by enhancing the antiapoptotic actions of these proteins [83]. Diraison et al. showed that, in *β*-cells, glucose regulates the expression of TCTP and its translocation from the cytosol to the nucleus by phosphorylation and glucose levels influence the sensitivity of *β*-cells to apoptosis. Palmitate treatment of *β*-cells decreased TCTP, resulting in increased *β*-cell death, and overexpression of TCTP prevented *β*-cell death. These authors also demonstrated that TCTP partially translocates to the mitochondrion in response to glucose. But it is not clear how glucose regulates TCTP translocation to various organelles and protects cells from apoptotic stimuli [84]. 

In addition, as described previously, FFA-induced activation of CHOP and JNK pathways decreased expression and increased the phosphorylation of Bcl-2, respectively [63, 76, 85, 86]. Phosphorylated Bcl-2 also enhances Ca^2+^ efflux from the ER and increases Ca^2+^ uptake by mitochondria [87]. Indeed, overexpression of Bcl-2 can influence the distribution of Ca^2+^ within the ER and mitochondria and can protect against apoptosis [88–90]. Together, these findings suggest the existence of a novel pathway involving the ER and mitochondria, through which these organelles orchestrate the regulation of death signals. In this complex scenario, further understanding of this link should give insights valuable for the identification of therapeutic targets to protect *β*-cell function and prevent type II diabetes.

## 7. Therapeutic Agents Targeted to the ER

ER stress has been found to be associated with obesity, insulin resistance, and type II diabetes. Therefore, agents that reduce ER stress are useful in treating obesity, peripheral insulin resistance, and hyperglycemia and type II diabetes. These agents reduce or prevent ER stress by improving the folding or processing capacity of the ER. Chemical chaperones such as PBA and TUDCA have been shown to regulate ER stress and improve insulin sensitivity *in vivo* [91]. In addition, salubrinal, a selective inhibitor of eif2*α* dephosphorylation, has been proposed as a novel therapy for diabetes. Salubrinal blocks eif2*α* dephosphorylation mediated by herpes simplex virus protein and inhibits viral replication [92]. But, in *β*-cells, selective inhibition of eif2*α* was found to potentiate lipotoxicity [27]. 

A growing body of evidence suggests that glucagon-like peptide 1 (GLP-1) and its analogues ameliorate experimental diabetes and preserve *β*-cell mass, protecting *β*-cells from apoptosis [93–95]. Activation of GLP-1 receptor by exendin-4 was shown to improve the survival of *β*-cells exposed to chemically induced ER stress via increased ATF4-CHOP expression. Interestingly, upon exposure to lipotoxic conditions, GLP-1 receptor activation prevented *β*-cell apoptosis by increasing cellular defense through the induction of Bip/GPR78 and the antiapoptotic protein junB [63]. 

Recently, in a search for novel therapeutic agents, plant-derived flavonoids were found to exhibit a broad spectrum of bioactivities. They display a remarkable array of biochemical and pharmacological actions, which may significantly affect the functions of various cellular systems [96]. Among flavonoids, methoxyflavonoids have beneficial hypolipidemic effects and suppress ER stress both *in vivo* and *in vitro* [97, 98]. In addition, quercetin, a flavonoid found in many plants, protects *β*-cells from oxidative damage and preserves *β*-cell integrity [99, 100]. Several studies have demonstrated that the isoflavone genistein, isolated from legumes, has antidiabetic effects presumably mediated by hypolipidemic effects, thereby increasing insulin sensitivity. Administration of genistein to animal and human islets increased islet proliferation, survival, and mass, mediated by activation of cAMP/PKA/ERK1/2 phosphorylation [101–103]. To date, the beneficial activities of flavonoids have been attributed mainly to their antioxidant properties. Detailed studies are needed to reveal the mechanisms beyond antioxidant activity underlying the beneficial effects of flavonoids on the ER-mitochondrial connection in the apoptotic cascade.

## 8. Conclusion

It is known from experimental evidence that ER stress contributes to tissue dysfunction and damage caused by glucolipotoxicity in diabetes. However, the mechanisms underlying *β*-cell dysfunction and death in diabetes are complex. Understanding this complex scenario and the agents that modulate the ER stress response to *β*-cell resistance to lipotoxic stress could have considerable impact on the treatment of *β*-cell failure and type II diabetes. Thus, additional studies are required to determine the link between ER stress and *β*-cell failure. This may potentially lead to the development of novel therapeutics to prevent type II diabetes.
